# Predicting cognitive decline: Which is more useful, baseline amyloid levels or longitudinal change?

**DOI:** 10.1016/j.nicl.2023.103551

**Published:** 2023-12-15

**Authors:** Gengsheng Chen, Nicole S. McKay, Brian A. Gordon, Jingxia Liu, Nelly Joseph-Mathurin, Suzanne E. Schindler, Jason Hassenstab, Andrew J. Aschenbrenner, Qing Wang, Stephanie A. Schultz, Yi Su, Pamela J LaMontagne, Sarah J. Keefe, Parinaz Massoumzadeh, Carlos Cruchaga, Chengjie Xiong, John C. Morris, Tammie L.S. Benzinger

**Affiliations:** a Departments of Radiology, Washington University in St. Louis School of Medicine, St. Louis, MO, USA; b Knight Alzheimer's Disease Research Center, Washington University in St. Louis School of Medicine, St. Louis, MO, USA; c Hope Center for Neurological Disorders, Washington University in St. Louis School of Medicine, St. Louis, MO, USA; d Department of Surgery, Washington University in St. Louis School of Medicine, St. Louis, MO, USA; e Department of Neurology, Washington University in St. Louis School of Medicine, St. Louis, MO, USA; f Department of Psychiatry, Washington University in St. Louis School of Medicine, St. Louis, MO, USA; g Divison of Biostatistics, Washington University in St. Louis School of Medicine, St. Louis, MO, USA; h Department of Neurology Massachusetts General Hospital, Harvard Medical School, Boston, MA, USA; i Banner Alzheimer’s Institute, Phoenix, AZ, USA

**Keywords:** Amyloid, PET, Cognition, Alzheimer’s disease, Longitudinal study

## Abstract

The use of biomarkers for the early detection of Alzheimer’s disease (AD) is crucial for developing potential therapeutic treatments. Positron Emission Tomography (PET) is a well-established tool used to detect β-amyloid (Aβ) plaques in the brain. Previous studies have shown that cross-sectional biomarkers can predict cognitive decline (Schindler et al.,2021). However, it is still unclear whether longitudinal Aβ-PET may have additional value for predicting time to cognitive impairment in AD. The current study aims to evaluate the ability of baseline- versus longitudinal rate of change in-^11^C-Pittsburgh compound B (PiB) Aβ-PET to predict cognitive decline. A cohort of 153 participants who previously underwent PiB-PET scans and comprehensive clinical assessments were used in this study. Our analyses revealed that baseline Aβ is significantly associated with the rate of change in cognitive composite scores, with cognition declining more rapidly when baseline PiB Aβ levels were higher. In contrast, no signification association was identified between the rate of change in PiB-PET Aβ and cognitive decline. Additionally, the ability of the rate of change in the PiB-PET measures to predict cognitive decline was significantly influenced by APOE ε4 carrier status. These results suggest that a single PiB-PET scan is sufficient to predict cognitive decline and that longitudinal measures of Aβ accumulation do not improve the prediction of cognitive decline once someone is amyloid positive.

## 1 Introduction

There are at least 5 million people currently living with age-related dementia in the United States, posing a major public health problem. Alzheimer’s disease (AD) is the most common form of dementia, accounting for about 70 % of all dementia cases in the elderly (Alzheimer's Association, 2016). AD care is expensive for the medical and social services sector, as well as the entire US economy. In contrast to the decreasing death rate of other major diseases over recent years, such as cardiovascular disease, the proportion of death attributed to AD has substantially increased, and it is now the third leading cause of death in the United States (Alzheimer's Association, 2016).

Previous research has highlighted that AD has a long preclinical phase lasting 10–20 years (Vermunt et al., 2019). This preclinical phase is characterized by an absence of overt clinical symptoms despite an accumulation of pathological changes within the brain. These changes typically begin with the accumulation of β-amyloid (Aβ), followed by the accumulation of pathological tau, structural disturbances in grey- and white-matter, and finally, a decline in cognition (Jack et al., 2010, Jack et al., 2013). Some cognitive decline may occur during this preclinical phase, but not an extent that disrupts an individual’s daily routine (Price and Morris, 1999). It has been proposed that the acceleration of cognitive decline may begin months, or even years, before individuals are normally diagnosed with AD-related dementia (Karr et al., 2018). Given that the pathological changes associated with AD begin years prior to cognitive symptoms, there is an increasing need to develop predictors of AD for use in the asymptomatic and early clinical phases.

In this study, we utilized the Washington University Knight Alzheimer's Disease Research Center (ADRC) dataset, which is dedicated to gathering data on preclinical participants, the majority of whom exhibit normal cognitive function at the baseline (Clinical Dementia Rating [CDR]® = 0). Our primary objective was to integrate imaging biomarker data with cognitive testing data to detect the earliest signs of cognitive decline in individuals with preclinical AD. There is a pressing need amongst AD researchers and clinicians to develop an optimal approach for the identification of individuals who are at the highest risk of progressing from a state of cognitive normalcy to mild cognitive impairment (MCI), and subsequently to AD-related dementia. Notably, the recent approval by the US FDA of two disease-modifying monoclonal antibody (mAb) treatments, which have proven effective in the very early stages of the disease, underscores the urgency of this area of neuroscience research. There is a compelling need to develop tools that aid in the identification of AD during its preclinical phase, ensuring that patients can access treatment at the earliest possible juncture.

Focusing on the earliest measurable pathological changes in preclinical AD, Aβ plaque deposition, previous studies have utilized PET for detecting and quantifying the presence of Aβ. For example, researchers have shown that Aβ-specific PET tracers, such as ^11^C-Pittsburgh compound B (PiB), can be used to detect Aβ plaques and measure the rate of Aβ accumulation over time throughout the disease process, including in the preclinical stage (Vlassenko et al., 2012, Villemagne et al., 2013, Villemagne et al., 2017). Recent studies have hypothesized that Aβ-PET scans could be useful for understanding the relationship between Aβ and cognition (Wang et al., 2018, Fodero-Tavoletti et al., 2009, Farrell et al., 2017). However, researchers must carefully consider the limitations of PET when considering whether baseline Aβ measurements are more sensitive to cognitive deterioration to avoid unnecessary repeated scans. Importantly, PET imaging requires patients to be exposed to ionizing radiation and the performance of multiple PET scans does pose risks to individuals, although these risks are very low (Brix et al., 2009, Nievelstein et al., 2012). Therefore, we were interested in evaluating whether a single measure of Aβ was better predictors of eventual cognitive decline, as compared to longitudinal measures of Aβ. We used a random coefficient model to evaluate the relationship between PIB-PET and cognition measures statistically in a cohort of individuals participating in research studies at the Charles F. and Joanne Knight Alzheimer Disease Research Center (Knight ADRC). This statistical method allows us to accommodate heterogeneous numbers of visits and intervals between visits, for participants in this study. Given that sporadic AD does not have a predetermined “estimated age of onset” and the rate of cognitive decline varies between subjects, it is difficult to determine when biomarker changes are occurring relative to the onset of dementia. These factors justify the use of a random coefficient approach, as this allows us to model separate time until dementia onset, and rates of change, for each individual. By including model terms that accommodate variability in these characteristics of dementia onset, we are able to increase the validity of conclusions drawn from these analyses.

## 2 Methods

### 2.1 Participants

Participants were enrolled in longitudinal studies of memory and aging at Knight ADRC at Washington University in St. Louis. Data was obtained through the Open Access Series of Imaging Studies (OASIS; https://www.oasis-brains.org) (Marcus et al., 2010). Individuals were excluded from analyses if they presented with dementia that was not primarily caused by AD, had active neurologic or psychiatric illness, had a history of serious head injury or clinically meaningful stroke, or used psychoactive drugs. Participants were required to have at least two longitudinal clinical and cognitive assessments and longitudinal Aβ-PET scans, resulting in a sample of 153 participants.

### 2.2 Clinical and cognitive assessment

In addition to PET imaging, each individual underwent clinical and cognitive assessments. The presence and severity of dementia was determined by experienced clinicians using the clinical dementia rating® (CDR®; Morris, 1993). A CDR of 0 indicates absence of dementia, and ratings of 0.5, 1, 2, and 3 indicate very mild, mild, moderate, and severe dementia, respectively. Each cohort at the Knight ADRC receives slightly different cognitive batteries, so only tests across all cohorts were considered in this study. A cognitive composite score was calculated from the average z-scores of 10 neuropsychological tests (Logical Memory, Logical Memory Delayed tests (Wechsler, 1997), Digit Span Forward, Digit Span Backward Tasks (Wechsler and Stone, 1973), Boston Naming Test (Kaplan et al., 1976), two subsets of the Trail Making Test: Part A and Part B (Armitage, 1945), two category fluency tasks: The Vegetable Naming and Animal Naming tests (Goodglass and Kaplan, 1983), and subscales of the revised Wechsler Adult Intelligence Scale (WAIS): Information and Block tests (Wechsler, 1997).

### 2.3 PET acquisition and processing

For the OASIS dataset, dynamic PIB PET scans were acquired on a Siemens Biograph 40 PET/CT or a Siemens/CTI EXACT HR + scanner for 60 min after tracer administration and reconstructed using standard iterative methods, with attenuation and scatter correction. All PET scans were processed with the PET Unified Pipeline (PUP, https://github.com/ysu001/PUP) using FreeSurfer derived ROIs (Su et al., 2018, Su et al., 2013) to calculate the standardized uptake value ratio (SUVR), using the cerebellar cortex as a reference region. Quantitative PET analysis used peak time windows of 30 to 60 min’ post-injection for PiB (Fischl, 2012, Su et al., 2013). Partial volume correction was performed using a geometric transfer matrix (Rousset et al., 1998, Su et al., 2015). To measure amyloid burden, we calculated the mean cortical SUVR (mcSUVR) by averaging the partial volume corrected SUVRs from the FreeSurfer ROIs in the lateral orbitofrontal, medial orbitofrontal, rostral middle frontal, superior frontal, superior temporal, middle temporal, and precuneus regions, as previously defined (Su et al., 2013). It is necessary to standardize methods for data collection and analysis to better aid cross-center, multi-tracer utility. The Centiloid Project was initiated to derive a standardized quantitative amyloid imaging measurement scale based upon normalization of data from the 18F-tracers to that of PiB. In this linear scale, young controls (≤45 years) have a mean of zero centiloid units (CL) and typical mild to moderate AD patients score on average 100CL (Klunk et al., 2015). In this study, we used the centiloid derived from mcSUVR.

### 2.4 Genotyping

Genomic DNA was isolated from peripheral blood samples using standard procedures. *APOE* genotyping was performed as described in previous work (Talbot et al., 1994).

### 2.5 Statistical analysis

We used a random coefficient model to statistically evaluate the relationship between PIB-PET and cognition measures. This statistical method allows us to accommodate heterogeneous numbers of visits and intervals between visits for participants in this study. This is an important feature because it increases the ecological validity of our conclusions, given that real-world patients are unlikely to have the same number of PET scans collected or the same time interval between scans. First, we were interested in quantifying how well baseline Aβ predicts cognitive performance. In order to accomplish this, a one-step random coefficient model was used (equation (1). In this model, time is set as a continuous variable (measured in years), representing the interval between the baseline cognitive assessment and each subsequent visit. Within this model, time is treated as both a fixed and random effect.

(1) Cognition=β1∗time+β2∗baselineAβ+β3*time∗baselineAβ+β4∗age+β5∗sex+β6∗education

In order to examine if longitudinal Aβ predict cognitive decline, a separate two-step random coefficient model was conducted. The first step of this model involved calculating the rate of change in Aβ (slope), treating time as both a random and a fixed effect (equation (2). The second step of this model quantified whether the rate of change in Aβ levels (slope) was predictive of longitudinal cognitive decline (equation (3)).

(2) Amyloid=β1∗time+β2∗age+β3∗sex+β4∗education

(3) Cognition=β1∗time+β2∗Aβslope+β3∗time∗Aβslope+β4∗age+β5∗sex+β6∗education

By adding a three-way interaction term to equation 3, mixed models were also used to determine the effect of *APOE* ε4 carrier status (Aβ slope*time*APOE ε4 carrier status) and sex (Aβ slope*time*sex) on modifying the effect of longitudinal Aβ on cognitive decline.

Last, we examine cognitive changes over time for three different Centiloid levels: low (Centiloid = 0), medium (Centiloid = 50), and high (Centiloid = 100). Models were fitted for cognition as a function of time and baseline Aβ, using mixed model output to obtain three different equations (Centiloid = 0, 50, 100).

All statistical analyses were performed using PROC MIXED in SAS Version 9.4 (SAS Institute Inc., Cary, NC), and *p* < 0.05 was regarded as statistically significant.

## 3 Results

Demographic characteristics of the participants at baseline are presented in Table 1. There were 153 participants in the study cohort, with an average age of 69.9 ± 5.9 years and average years of education of 15.5 ± 2.5. Of the 153 participants, 87 (56.8 %) identified as female and 52 (34 %) were *APOE* ε4 carriers. Among the APOE ε4 carriers, 62 % are female. Participants were followed for an average of 6.01 ± 2.0 years’ assessments. The mean cognitive composite score was 0.37 ± 0.42. The baseline scores and main results for the individual cognitive tests are presented in Supplementary Tables S1 and S2, respectively.

**Table 1:** Baseline demographics table.

Variable	N = 153
Age(year), mean ± SD	69.97 ± 5.95
Female, n (%)	87(56.8)
Years of education, mean ± SD	15.5 ± 2.47
Follow-up (year), mean ± SD	6.01 ± 1.96
Cognitive composite score, mean ± SD	0.37 ± 0.42
APOE ε4 carrier, n (%)	52 (34)
APOE ε4 female carrier, n (%)	32 (62)
CDR > 0, n (%)	12(7.8)

### 3.1 Aβ derived from a single PET image predicts cognitive decline

Our first analysis aimed to characterize whether Aβ derived from a single baseline PET scan could accurately predict cognitive decline. Our results showed that baseline Aβ significantly predicts the rate of change in cognitive composite score (*p* = 0.029, t = -2.2, Table 2**, see** Table S2 **for detail for the individual tests.**). For all participants, older individuals (*p* < 0.0001), males (*p* < 0.0001), individuals with less education (*p* < 0.0001) had significantly lower baseline performance on the cognitive composite.

**Table 2:** Baseline Aβ prediction of cognition decline.

Predictors	DF	Estimate	t-value	*p*-value
Time	148	−0.01435	−1.94	0.0549
Baseline-Aβ	638	−0.00168	−1.77	0.0774
Baseline-Aβ*time	638	−0.00046	−2.20	0.029
Age	638	−0.026	−5.05	<0.0001
Sex	638	−0.27	−4.63	<0.0001
Education	638	0.055	4.47	<0.0001

Baseline_Aβ *time: Baseline Aβ predicts cognition decline.

**Fig. 1:**
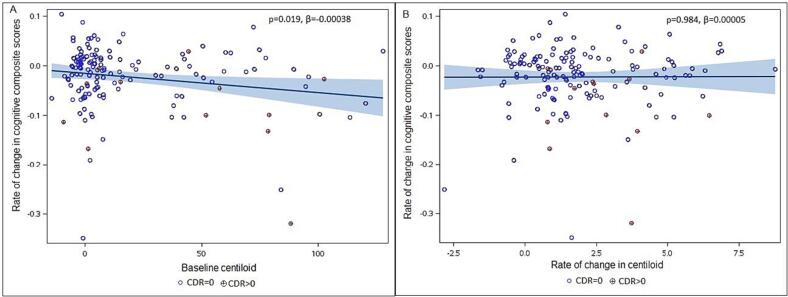
(**A**) Association between rate of change in cognition and baseline Aβ levels. The X-axis is the baseline Aβ, and Y-axis reflects rate of change in cognitive composite scores; (**B**) Association between rate of change in cognition and rate of change in Aβ levels. Red symbols are CDR > 0 group and blue symbols are CDR = 0 group. (For interpretation of the references to colour in this figure legend, the reader is referred to the web version of this article.)

We generated a scatter plot to investigate the relationship between baseline Aβ and cognitive decline. This analysis revealed a significant association between the rate of change in cognitive composite score and baseline Aβ (*p* = 0.019, estimate β = -0.00038, Fig. 1**A**).

### 3.2 Rate of changes in Aβ derived from longitudinal PET scans does not predict cognitive decline

Building from the prior analyses, we were also interested in characterizing how well the rate of change in Aβ, derived from longitudinal PET scans, predicts cognitive decline. Longitudinal Aβ was not significantly associated with the rate of change in cognitive composite score (*p* = 0.917, t = 0.08, Table 3). We also generated a scatter plot to characterize the relationship between rate of change in Aβ and cognitive decline. Fig. 1**B** shows that rate of change in Aβ is not significantly associated with the rate of change in the cognitive composite score (*p* = 0.984, estimate β = 0.00005, Fig. 1**B**).

**Table 3:** Longitudinal Aβ prediction of cognition decline.

Predictors	DF	Estimate	t-value	*p*-value
Time	148	−0.023	−2.6	0.01
Aβ-slope	638	−0.004	−0.28	0.782
Aβ-slope*time	638	0.000339	0.10	0.917
Age	638	−0.026	−5.33	<0.0001
Sex	638	−0.27	−4.45	<0.0001
Education	638	0.054	4.36	<0.0001

Aβ_slope*time: Rate of change in Aβ predicts cognition decline.

### 3.3 Impact of covariates on the relationship between longitudinal Aβ and cognitive change

Finally, we examined the impact of important covariates on the relationship between longitudinal Aβ and cognitive change. Table 4 shows that the presence of the *APOE* ε4 allele increases AD risk by accelerating cognitive decline (*p* = 0.028, t = 2.2). Sex was found to have no significant effects on the association between amyloid with cognitive decline (*p* = 0.088, t = -1.7).

**Table 4:** Impact of APOE ε4 carrier status and sex on longitudinal Aβ prediction of cognitive decline.

Predictors	DF	Estimate	t-value	*p*-value
Slope*sex*time	784	−0.00606	−1.7	0.0888
Slope* APOE ε4 *time	784	0.008071	2.2	0.0279
Age	784	−0.029	−4.92	<0.0001
Sex (ref = male)	784	0.2314	2.27	0.023
Education	784	0.058	3.97	<0.0001

### 3.4 Comparing the baseline and rate of change in Aβ

**Fig. 2:**
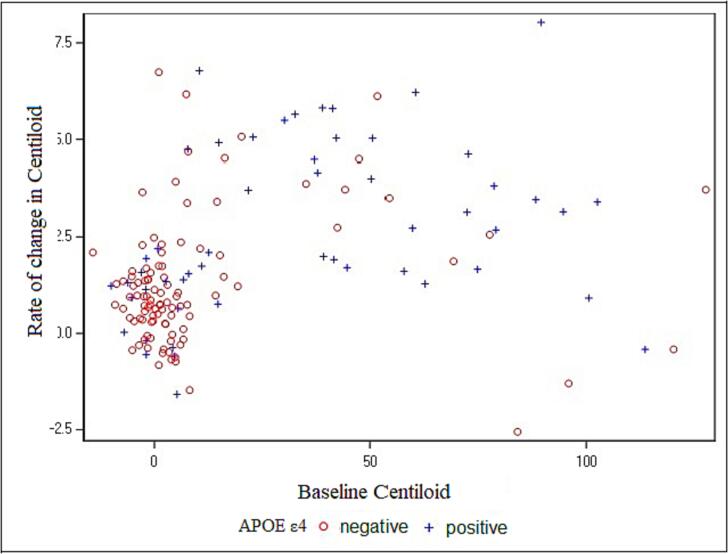
Scatter plot depicting the relationship between baseline Aβ levels and rate of change in Aβ levels. The Y-axis is rate of change in Aβ levels; the X-axis is the baseline Aβ levels. Red symbols are the APOE ε4 negative group and blue symbols are APOE ε4 positive group. (For interpretation of the references to colour in this figure legend, the reader is referred to the web version of this article.)

In order to understand why the baseline centiloid is a better predictor of cognitive decline than longitudinal centiloid, we plotted the relationship between these measures using a scatterplot. Fig. 2 shows that the rate of centiloid was low when baseline centiloid was also low, while accumulation rates increased to a maximum when baseline centiloidwas around 50. Accumulation rates declined above this maximum, approaching zero when baseline centiloid was greater than 100.

**Fig. 3:**
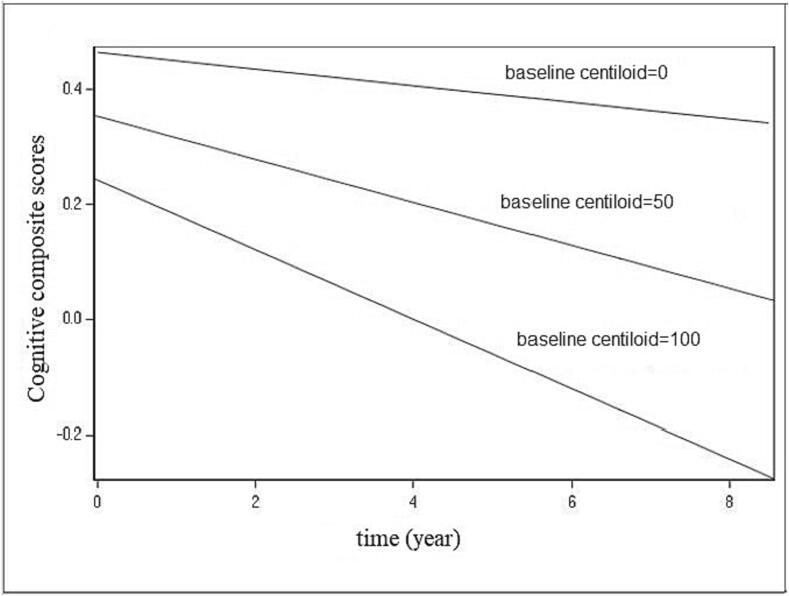
Cognitive composite scores change over time (year) for different baseline centiloid. Time is a continuous variable (measured in years), representing the interval between the baseline cognitive assessment and each subsequent visit.

Given this inverted U finding, we were motivated to predict cognition changes over time for three different baseline centiloid levels. Fig. 3 shows that cognition decreases with time for the three different baseline centiloid levels examined. More specifically, scores on the cognitive composite score decrease with time for different levels of baseline centiloid, when baseline centiloid increases, cognition decreases more rapidly.

## 4 Discussion

We aimed to investigate the relationship between Aβ and cognition in the preclinical and early impairment stages of AD. More specifically, we compared the utility of a single baseline measure of Aβ versus longitudinal measures of Aβ for predicting cognitive decline, as defined by a cognitive composite score. This investigation revealed that baseline Aβ is a better predictor of cognitive decline than longitudinal Aβ. This is potentially due to the non-linear, inverted U-shaped relationship we observed between baseline and longitudinal PET measures (see Fig. 2). This finding is consistent with previous studies that also reported a U-shaped function between these two measures (Clifford et al., 2013; Mishra et al., 2018, Schindler et al., 2021). This inverted U-shaped pattern may indicate that the rate of change in Aβ may be less informative at all points in the disease course than overall levels of pathology. Importantly, the values of baseline Aβ are much greater than the absolute values for longitudinal change in Aβ, and thus, longitudinal Aβ changes can be easily affected by signal noise. Together, these results suggest that baseline Aβ is more accurate than longitudinal Aβ for predicting cognitive decline. This holds significant practical implications, as undergoing multiple PET scans necessitates patients being repeatedly subjected to the potential risks of exposure to ionizing radiation (Brix et al., 2009, Nievelstein et al., 2012). Thus, it is imperative to explore whether conducting multiple amyloid PET scans on individuals with initial amyloid positivity is clinically necessary or beneficial. Our findings support the concept that a single amyloid PET scan may be adequate for evaluating dementia risk in the context of AD. This has substantial ramifications for public health, particularly in the current era of AD treatment with monoclonal antibodies (mAbs), as it has the potential alleviate the burden on patients and healthcare systems through earlier diagnosis and treatment of dementia.

Several previous studies have also investigated the relationship between AD biomarkers and subsequent cognitive changes in individuals initially classified as cognitively normal. For instance, a recent study by Hanseeuw and colleagues revealed a link between amyloid accumulation and tau accumulation, with subsequent associations with the rate of cognitive decline also observed (Hanseeuw et al., 2019). However, this study did not establish a direct relationship between longitudinal Aβ levels and cognition. Another study suggested that Aβ load is predictive of future cognitive decline, but this study solely investigated how baseline amyloid predicts cognitive decline with no longitudinal analyses performed (Dumurgier et al., 2017). The novelty of our study lies in our comparative evaluation of baseline and longitudinal amyloid to determine which holds greater value for the prediction of cognitive outcomes. Given the cost and radiation exposure risks associated with PET scans, it is essential to ascertain whether a single scan is sufficient for the early detection of the disease.

In our current study, we also illustrated our model fit on cognitive change over time for three different amyloid levels, defined by low, medium, and high centiloid units, using equation (1) and the parameters obtained from the model. Our results show that cognition decreased over time and that when baseline Aβ is higher, cognition decreases more rapidly. This finding is consistent with a recent paper (Farrell et al., 2017), which also reported that higher baseline Aβ burden predicted a steeper decline in episodic memory, processing speed, vocabulary, and Mini-Mental State Examination performance. Furthermore, another study by Lim et al (Lim et al., 2013) focusing on Aβ-positive adults combined cognitively normal participants with those with mild cognitive impairment and dichotomizing them into low- and high-Aβ groups. They reported a greater memory decline for the group with higher Aβ levels compared to the group with lower Aβ levels. These findings suggest that the magnitude of Aβ burden may be useful in predicting the rate of future cognitive decline, with those with the greatest burdens showing the most decline.

Our study also reported that important covariates, such as the presence of the *APOE* ε4 allele, can significantly increases AD risk by accelerating amyloid deposition, which is consistent with some previous studies (Liu et al., 2019). One recent study suggested that longitudinal Aβ measures in a group of *APOE* ε4 negative individuals were significantly predictive of cognitive decline (Hsiung et al., 2004, Paranjpe et al., 2019). Furthermore, these authors also found that in individuals with at least one copy of the *APOE* ε4 allele, the rate of change in Aβ is not a significant predictor of cognitive decline. This may be because APOE ε4 status will play a primary role in predicting cognitive decline in individuals who carry an APOE ε4 allele, but when this allele is absent, Aβ becomes the primary predictor of cognitive decline. This could also be because Aβ levels increase earlier in individuals who are APOE e4 carriers, whereas Aβ does not increase until later in APOE e4 negative individuals (Schindler et al., 2021).

The use of a random coefficient model (RCM) is a major statistical strength of this study, as this approach enables the use of different number of visits and visit intervals for each individual across variables of interest in our analyses. Most previous work in this field focused on using general linear mixed models (LMM), which assume a homogenous number of visits and inter-visit intervals for all study participants ( Lawrence et al., 2018; Shokouhi et al., 2013). The patient cohort for this study had highly variable numbers of visits and inter-visit intervals between participants, with some individuals attending only two visits, while others had up to seven, precluding the use of LMM for our analyses. Thus, we adopted an RCM approach in this study to examine the individual-level variation in the relationship between predictor variables (e.g., rate of change in amyloid) and outcomes (e.g., cognition). LMM has notable restrictions that also render it unsuitable for this study, such as its requirement for linear disease progression during the follow-up period, which is unlikely in an AD patient cohort. In addition, our two-step model is very intuitive and simple, compared to complex existing models used in previous longitudinal studies. Patients outside of research settings are very unlikely to have homogenous numbers of clinical visits or consistent intervals between visits, thus, a random coefficient model is better able to statistically control for this expected variability. By using this approach, we are confident that our conclusions are as rigorous, reproducible, and clinically relevant as possible, facilitating their translation towards clinical use.

Despite the major advantages of our study, there are some limitations to note. For example, the Aβ level used in this study is an average of measures across several critical brain regions. This measure was chosen as it is standard in this field, however, future iterations of this work should investigate Aβ levels across a wider number of regions in order to fully characterize the relationship of interest. Next, there are some limitations to our two-step model as estimated slopes, which are used for predictive calculations, are subject to errors. But our two-step model is very intuitive and simple, comparing to existing complex models used in previous longitudinal studies. Finally, this project only included PiB-PET as a potential biomarker of cognitive decline, as a way to better mimic potential clinical data, which is often limited to single biomarker. In order to fully understand what biological changes are best predictive of the eventual cognitive decline associated with AD, it may be helpful in future analyses to include a wider selection of relevant biomarkers, such as volumetric Magnetic Resonance Imaging (MRI), tau PET, or fluid biomarkers.

In summary, the current study provides evidence that baseline Aβ is more accurate than longitudinal Aβ for predicting cognition decline. Importantly, this study also confirms that covariates, such as the presence of the *APOE* ε4 allele, have deleterious group level effects on the ability of longitudinal Aβ measures to predict cognitive decline. From a clinical point of view, these results are encouraging as they provide evidence that only one amyloid positive PET scan is necessary in order to make predictions about future cognitive decline in individuals at risk for developing AD. This is especially important, as PET imaging is a limited resource that will be difficult to scale to large clinical populations, and requires the use of radioactive radiotracers, so minimizing the exposure to these for patients is an important step forward for understanding and treating AD. Finally, given that PET is able to image one of the earliest pathological changes known to occur in the preclinical phase of AD, Aβ deposition, our study provides strong support for the use of this technology for early identification of those who are most at risk of developing AD-related dementia.

## CRediT authorship contribution statement

**Gengsheng Chen:** . **Nicole S. McKay:** Writing – review & editing. **Brian A. Gordon:** Conceptualization, Methodology, Writing – review & editing. **Jingxia Liu:** Methodology, Writing – review & editing. **Nelly Joseph-Mathurin:** Writing – review & editing. **Suzanne E. Schindler:** Writing – review & editing. **Jason Hassenstab:** Writing – review & editing. **Andrew J. Aschenbrenner:** Writing – review & editing. **Qing Wang:** Writing – review & editing. **Stephanie A. Schultz:** Writing – review & editing. **Yi Su:** Writing – review & editing. **Pamela J LaMontagne:** Data curation. **Sarah J. Keefe:** Data curation. **Parinaz Massoumzadeh:** Writing – review & editing. **Carlos Cruchaga:** Writing – review & editing. **Chengjie Xiong:** Methodology. **John C. Morris:** Data curation, Funding acquisition, Project administration. **Tammie L.S. Benzinger:** Conceptualization, Funding acquisition, Investigation, Project administration, Resources, Supervision, Writing – review & editing.

## Declaration of competing interest

The authors declare the following financial interests/personal relationships which may be considered as potential competing interests: Dr. Benzinger has held investigator-initiated research funding from the NIH, the Alzheimer’s Association, the Barnes-Jewish Hospital Foundation, Siemens Healthiness and Avid Radiopharmaceuticals (a wholly owned subsidiary of Eli Lilly). Dr. Benzinger participates as a site investigator in clinical trials sponsored by Avid Radiopharmaceuticals, Eli Lilly, Biogen, Eisai, Jansen, and Roche. Dr. Benzinger performs paid and unpaid consulting for Biogen, Eli Lilly, Eisai, Roche and Siemens. SES is analyzing biomarker data provided by C2N Diagnostics to Washington University.

## Data Availability

Data will be made available on request.
