# Supplementary material for: Predicting cognitive decline: Which is more useful, baseline amyloid levels or longitudinal change?
Source: Neuroimage Clin. 2023 Dec 15;41:103551. doi: 10.1016/j.nicl.2023.103551 (PMC10788301; doi:10.1016/j.nicl.2023.103551)
Supplement: Supplementary data 1 [file mmc1.docx]

**Supplemental Material**

Table S1. Baseline neuropsychological test scores.

| Variable | Mean(SD) | | |
| --- | --- | --- | --- |
| Boston Naming Test | | 27.58(2.30) | |
| Trail Making Test – Part B | | 82.21(39.27) | |
| Digit Symbol Task | | | 55.44(12.11) |
| Animal Naming Test | | | 20.56(5.79) |
| Trail Making Test – Part A | | | 30.83(10.33) |
| Vegetable Naming Test | | | 14.18(4.17) |
| Digit Span Backward-trials correct | | | 6.65(1.95) |
| Digit Span Backward-length | | | 4.8(1.07) |
| Digit Span Forward- trials correct | | | 8.43(2.01) |
| Digit Span Forward-length | | | 6.68(1.08) |
| Logical Memory | | | 13.2(3.99) |

Table S2. Baseline and longitudinal Aβ predict cognition decline

| Variable | Aβ-slope*time | | Baseline-Aβ*time | |
| --- | --- | --- | --- | --- |
|  | *p*-value | Estimate | *p*-value | Estimate |
| Boston Naming Test | 0.563 | 0.014 | 0.039 | -0.003 |
| Trail Making Test – Part B | 0.592 | 0.183 | 0.058 | -0.041 |
| Digit Symbol Task | 0.932 | 0.005 | 0.075 | -0.007 |
| Animal Naming Test | 0.129 | -0.05 | 0.022 | -0.005 |
| Trail Making Test – Part A | 0.584 | 0.066 | 0.166 | -0.01 |
| Vegetable Naming Test | 0.189 | -0.034 | 0.015 | -0.004 |
| Digit Span Backward-trials correct | 0.416 | -0.01 | 0.520 | -0.0005 |
| Digit Span Backward-length | 0.662 | -0.003 | 0.501 | -0.0003 |
| Digit Span Forward- trials correct (WAIS-R) | 0.562 | 0.005 | 0.408 | -0.0005 |
| Digit Span Forward-length (WAIS-R) | 0.873 | -0.0009 | 0.347 | -0.0004 |
| Logical Memory (WMS-III) | 0.041 | 0.053 | 0.300 | -0.002 |

Aβ_slope*time: Rate of change in Aβ predicts cognition decline;

Baseline_ Aβ *time: Baseline Aβ predicts cognition decline.
